# Is simultaneous cranioplasty with cerebrospinal fluid shunts implantation as safe as staged procedures?

**DOI:** 10.3389/fneur.2022.995897

**Published:** 2022-10-10

**Authors:** Qian Zhou, Wei Shen, Zhiying Zhou, Xiaofeng Yang, Liang Wen

**Affiliations:** ^1^The First Affiliated Hospital, Zhejiang University School of Medicine, Hangzhou, China; ^2^Department of Neurosurgery, Beilun People's Hospital in Ningbo, Ningbo, China

**Keywords:** cranioplasty, ventriculoperitoneal shunts, postoperative complications, postoperative bleeding, postoperative infection

## Abstract

**Objective:**

The combination of cranioplasty and ventriculoperitoneal shunt is a therapeutic strategy for patients with hydrocephalus after decompressive craniectomy (DC). However, the efficacies of simultaneous vs. staged surgery in reducing postoperative complications have not been conclusively determined. This was a meta-analysis of relevant studies to assess whether simultaneous surgery significantly reduces postoperative complication risks, compared to staged surgery.

**Methods:**

We systematically searched PubMed, Embase, Cochrane, Web of science databases for studies (published by 11 May 2022) comparing patients undergoing concurrent and staged cranioplasty and ventriculoperitoneal shunt. Our main endpoints were; overall postoperative complications, postoperative bleeding, postoperative infection and reoperation. We assessed the pooled data using a random effects model to compare complication rates using odds ratios (ORs) and 95% confidence intervals (CIs).

**Results:**

Of the 494 identified studies, 12 were included in our analysis (*N* = 651 participants). Compared to staged surgery, concurrent surgery increased the relative risk for overall complications (pooled OR: 2.00; 95% CI: 1.10–3.67), however, it did not increase the relative risks for postoperative bleeding, postoperative infection or reoperation. Subgroup analysis revealed that in the Asian population, concurrent surgery increased the relative risks for overall complications (staged vs. concurrent group: OR: 2.41, 95% CI: 1.51–3.83, *I*^2^ = 0.0%) and postoperative infections (staged vs. concurrent group: OR: 2.35, 95% CI: 1.06–5.21, *I*^2^ = 31.8%).

**Conclusion:**

Compared to staged surgery, concurrent surgery increases the overall complication rates. However, differences between the two therapeutic approaches in terms of postoperative bleeding, postoperative infection, or reoperation are insignificant. Simultaneous surgery was associated with increased overall post-operative complications and post-operative infection rates in the Asian population.

## Introduction

Decompressive craniectomy (DC) is an important life-saving treatment option for patients with malignant intracranial pressure (ICP) elevations due to traumatic brain injury (TBI), malignant infarction, intracranial hemorrhage, and other neurological diseases. Although there are a range of complications following DC and cisternal drainage can be used as a complementary measure to decompressive craniectomy (1), DC remains the most widely used treatment for malignant ICP. Diseases and complications that lead to DC are often associated with abnormal CSF flows, and these primary diseases as well as DC surgery may result in long-term hydrocephalus. In a previous study, it was noted that 10–40% of patients undergoing DC developed hydrocephalus (2). Ventriculoperitoneal shunt (VPS) can provide a long-term diversion of the CSF and resolves hydrocephalus after DC. The rate of VPS implantation after DC is between 5 and 15% (3).

Patients with hydrocephalus after DC require cranioplasty and VPS implantation. However, the optimal sequence of these two procedures has not been established. Simultaneous VPS placement and cranioplasty can reduce costs and hospital length of stay for patients (4–6). In some reports, simultaneous placement of VPS with cranioplasty resulted in higher complication rates and longer hospital length of stay, compared to staged procedures (5–8). In contrast, other studies did not find significant differences between risk characteristics of infection and VPS malfunction when staged vs. concurrent VPS placement and cranioplasty were assessed (9).

Moreover, it has not been conclusively determined whether staged or concurrent surgery reduces surgical complications. One meta-analysis compared complication rates in patients undergoing concurrent vs. staged cranioplasty and ventriculoperitoneal shunts. Since the meta-analysis by Jung et al. was published 2 years ago, six new relevant studies have been published and incidences of complications from concurrent and staged surgery remain controversial. Therefore, we performed a meta-analysis to characterize and compare complication rates in patients subjected to concurrent and staged surgery.

## Methods

### Search strategy and selection criteria

This systematic review and meta-analysis is presented following the Preferred Reporting Items for Systematic Reviews and Meta-Analyses (PRISMA) statement.

We searched the PubMed, EMBASE, Web of Science and Cochrane Library databases to identify relevant studies published up to 11th May 2022. The MeSH terms and text used in the search were: “cranioplasty”, “ventriculoperitoneal shunt”, “hydrocephalus” and “decompressive craniectomy”. The complete search strategy in PubMed was: (((“Ventriculoperitoneal Shunt”[Mesh]) OR ((((((((Shunt, Ventriculoperitoneal) OR (Shunts, Ventriculoperitoneal)) OR (Ventriculoperitoneal Shunts)) OR (Ventriculo-peritoneal Shunt)) OR (Shunt, Ventriculo-peritoneal)) OR (Shunts, Ventriculo-peritoneal)) OR (Ventriculo peritoneal Shunt)) OR (Ventriculo-peritoneal Shunts))) AND ((Cranial vault reconstruction) OR (Cranioplasty))) AND (((((((Craniectomy, Decompressive) OR (Decompressive Craniectomies)) OR (Decompressive Craniotomy)) OR (Craniotomy, Decompressive)) OR (Decompressive Craniotomies)) OR (“Decompressive Craniectomy”[Mesh])) OR ((“Hydrocephalus”[Mesh]) OR (((((((((((((((((((((((((((Hydrocephaly) OR (Cerebral Ventriculomegaly)) OR (Cerebral Ventriculomegalies)) OR (Ventriculomegalies, Cerebral)) OR (Ventriculomegaly, Cerebral)) OR (Communicating Hydrocephalus)) OR (Hydrocephalus, Communicating)) OR (Congenital Hydrocephalus)) OR (Hydrocephalus, Congenital)) OR (Hydrocephalus Ex-Vacuo)) OR (Hydrocephalus Ex Vacuo)) OR (Hydrocephalus Ex-Vacuos)) OR (Obstructive Hydrocephalus)) OR (Hydrocephalus, Obstructive)) OR (Post-Traumatic Hydrocephalus)) OR (Hydrocephalus, Post-Traumatic)) OR (Post Traumatic Hydrocephalus)) OR (Aqueductal Stenosis)) OR (Aqueductal Stenoses)) OR (Stenoses, Aqueductal)) OR (Stenosis, Aqueductal)) OR (Fetal Cerebral Ventriculomegaly)) OR (Cerebral Ventriculomegalies, Fetal)) OR (Cerebral Ventriculomegaly, Fetal)) OR (Fetal Cerebral Ventriculomegalies)) OR (Ventriculomegalies, Fetal Cerebral)) OR (Ventriculomegaly, Fetal Cerebral)))). The other databases were searched as presented in Supplementary material.

### Study selection and data extraction

The inclusion criteria were: (i) Prospective or retrospective studies involving adult patients with decompressive craniectomy combined with hydrocephalus, comparing simultaneous cranioplasty and ventriculoperitoneal shunts with staged surgery, and reporting on post-operative complications in patients. The exclusion criteria were: (i) Studies that did not involve humans; (ii) Reviews, letters, and meeting records; (iii) Studies written in a language other than English; and (iv) Studies that did not evaluate concurrent cranioplasty and ventriculoperitoneal shunts vs. staged surgery. The assessed outcome was the number of patients with any post-operative complication episodes. Two reviewers (Zhou and Shen) independently reviewed the study titles and abstracts, and assessed the full text of studies that satisfied the inclusion criteria. The following data were extracted from each selected study: QZ, publication year and study design, study population and regions, sample size number, and number of participants with any post-operative complication episodes. Two independent reviewers (Zhou and Shen) assessed the risk of bias according to PRISMA recommendations.

### Statistical analysis

We compared postoperative complication incidences using a random effects model and the results expressed as dominance ratios (ORs) and 95% confidence intervals (CIs). The effects of cranioplasty and ventriculoperitoneal shunts on four outcomes were assessed: overall postoperative complications, postoperative bleeding, postoperative infection, and reoperation.

Heterogeneity among the studies were evaluated using the Cochran *Q-*test and the *I*^2^ statistic. If *I*^2^ > 50%, moderate to high heterogeneity was considered across studies, after which subgroup analyses were performed to identify potential heterogeneity sources.

In meta-analysis of each outcome, sensitivity analysis was limited to comparing infections, bleeding and reoperation rates after concurrent cranioplasty and ventriculoperitoneal shunts with staged surgery, which reduced heterogeneity in overall analysis in terms of outcome changes induced by different complications.

Publication bias was assessed by visual asymmetry in funnel plots and by Egger and Begg tests. *p* ≤ 0.1 denoted significant publication bias. The trim-and-fill method was used to estimate the effects of publication bias on interpretation of results. Analyses were performed using Stata 12.0 (StataCorp, College station, TX, USA). *p* ≤ 0.05 was set as the threshold for statistical significance.

## Results

**Figure 1:**
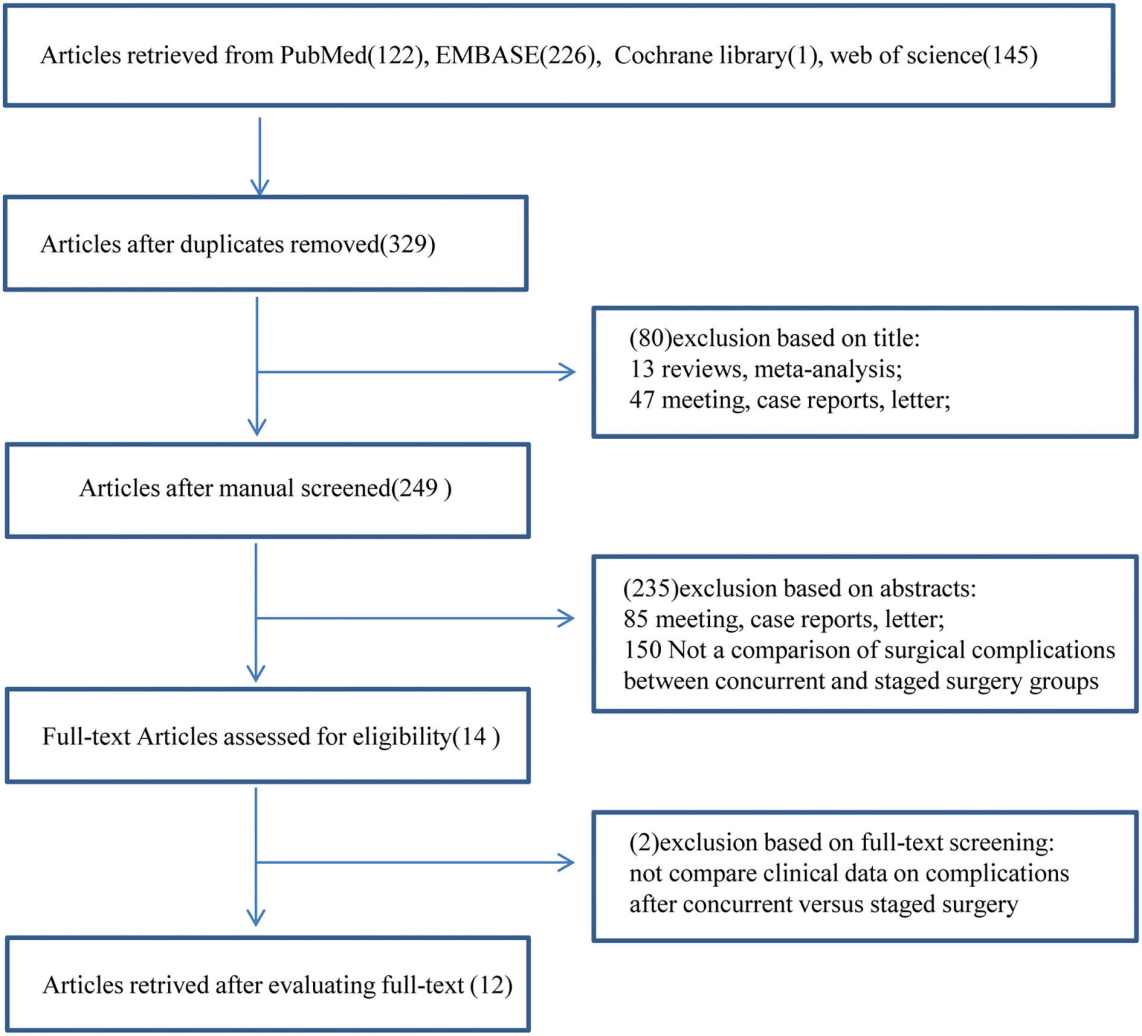
Flow diagram for selection of studies included in this meta-analysis.

We obtained a total of 494 studies in PubMed, EMBASE, Web of Science and the Cochrane Library databases. Among them, 165 duplicate studies were excluded. We excluded 80 studies after screening the titles and 235 studies after reviewing the abstracts. Then, we evaluated the remaining 14 full-text articles and excluded two studies that did not compare clinical data on complications after concurrent vs. staged surgery. In total, 12 studies were ultimately included in this study (Figure 1) (3, 5–17).

The 12 studies (comprising 651 participants) were all published between 2014 and 2022 (Table 1). They all reported on overall complications and post-operative infections after concurrent vs. staged surgery, nine reported on post-operative bleeding while four reported on re-operation. Of the 12 studies, four involved Caucasian populations, while the remaining eight studies focused on Asian populations. Characteristics of the 12 studies are summarized in Tables 1, 2.

**Table 1:** Baseline characteristics for studies included in the meta-analysis.

**Study**	**Study setting**	**N; Sex (male/** **female)**	**Mean age**	**Race**	**Trauma**	**Surgical strategy**		**CP with autobone**	**Programmable shunt**	**Complications**
						**Simultaneous**	**Staged**			
Ting et al. (10)	RO, single	49; (33/16)	NA	Asian	49	17	32	48	15	OC; Infection; IH; SE; RO
Meyer et al. (3)	RO, single	50; (29/21)	43	Caucasian	19	26	24	50	NA	OC; infection; SB
Zhou et al. (11)	RO, single	78; (54/24)	NA	Asian	52	15	63	1	76	OC; IH; infection; Epilepsy; SO; Shunt excess; Pneumonia;
Lin et al. (12)	RO, single	56; (35/21)	57.5	Asian	39	19	37	NA	NA	OC; infection; Shunt excess; RO
Heo et al. (6)	RO, single	51; (31/20)	NA	Asian	23	32	19	51	51	OC; SE; IH; CI
Jung et al. (13)	RO, single	24 (11/13)	53.1	Asian	15	14	10	24	1	OC; SE; IH; infection; SO; Sunken bone plate
Gill and Choi (14)	RO, single	81 (42/39)	61.3	Asian	29	18	63	79	81	OC; IH; infection; Pneumocephalus
Zhang et al. (15)	RO, single	86 (71/15)	NA	Asian	50	22	64	NA	66	OC; infection; IH; Epilepsy; SE; SSFS; SO
Rosinski et al. (9)	RO, single	40 (12/28)	49.4	Caucasian	NA	18	22	37	NA	OC; infection; CSF leak; DVT; PE; RO
Von der Brelie et al. (16)	RO, single	NA	NA	Caucasian	NA	10	27	NA	NA	OC; infection; Aseptic necrosis
Schuss et al. (5)	RO, single	41 (17/24)	NA	Caucasian	15	17	24	NA	NA	OC; IH; infection; others
Yang et al. (17)	RO, single	58 (46/12)	42.8	Asian	46	28	30	58	58	OC; IH; infection; RO

CP, cranioplasty; NA, not available; RO, retrospective observational; IH, Intracranial hematoma; SE, Subdural effusion; RO, Reoperation; SO, Shunt Obstruction; OC, overall complications; SSFS, Sinking skin flap syndrome; DVT, deep vein thrombosis; PE, pulmonary embolism.

**Table 2:** Summary of postoperative complications in different studies (simultaneous operation vs. staged operation).

**Study**	**Simultaneous vs. staged**				
	**Overall complications**	**Infections**	**Intracranial Hematoma**	**Reoperation**	**Others**
Ting et al. (10)	5/17 vs. 3/32	3/17 vs. 1/32	1/17 vs. 2/32	2/17 vs. 2/32	2/17 vs. 1/32
Meyer et al. (3)	4/26 vs. 7/24	3/26 vs. 4/24	NA	NA	1/26 vs. 3/24
Zhou et al. (11)	9/15 vs. 22/63	4/15 vs. 8/63	4/15 vs. 7/63	NA	1/15 vs. 7/63
Lin et al. (12)	3/19 vs. 5/37	1/19 vs. 5/37	NA	0/19 vs. 2/37	2/19 vs. 0/37
Heo et al. (6)	18/32 vs. 4/19	6/32 vs. 1/19	4/32 vs. 1/19	NA	8/32 vs. 3/19
Jung et al. (13)	9/14 vs. 7/10	3/14 vs. 3/10	3/14 vs. 1/10	NA	4/14 vs. 2/10
Gill and Choi (14)	6/18 vs. 7/63	2/18 vs. 7/63	3/18 vs. 0/63	NA	1/18 vs. 0/63
Zhang et al. (15)	8/22 vs. 18/64	5/22 vs. 3/64	0/22 vs. 3/64	NA	4/22 vs. 16/64
Rosinski et al. (9)	9/18 vs. 18/22	5/18 vs. 12/22	1/18 vs. 3/22	2/18 vs. 6/22	3/18 vs. 3/22
Von der Brelie et al. (16)	6/10 vs. 4/27	3/10 vs. 1/27	NA	NA	3/10 vs. 3/27
Schuss et al. (5)	8/17 vs. 3/24	7/17 vs. 0/24	1/17 vs. 1/24		0/17 vs. 2/24
Yang et al. (17)	6/20 vs. 5/38	5/20 vs. 1/38	0/20 vs. 2/38	4/20 vs. 1/38	NA

Infections: cranioplasty infection, VPS infection and wound infection. NA, not available.

**Figure 2:**
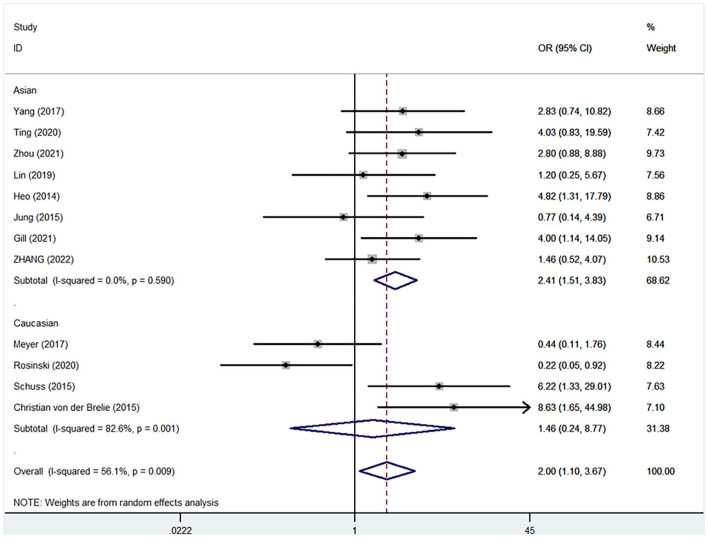
Comparisons of overall postoperative complications between staged and simultaneous cranioplasty and ventriculoperitoneal shunts. OR, odds ratio; CI, confidence interval.

**Figure 3:**
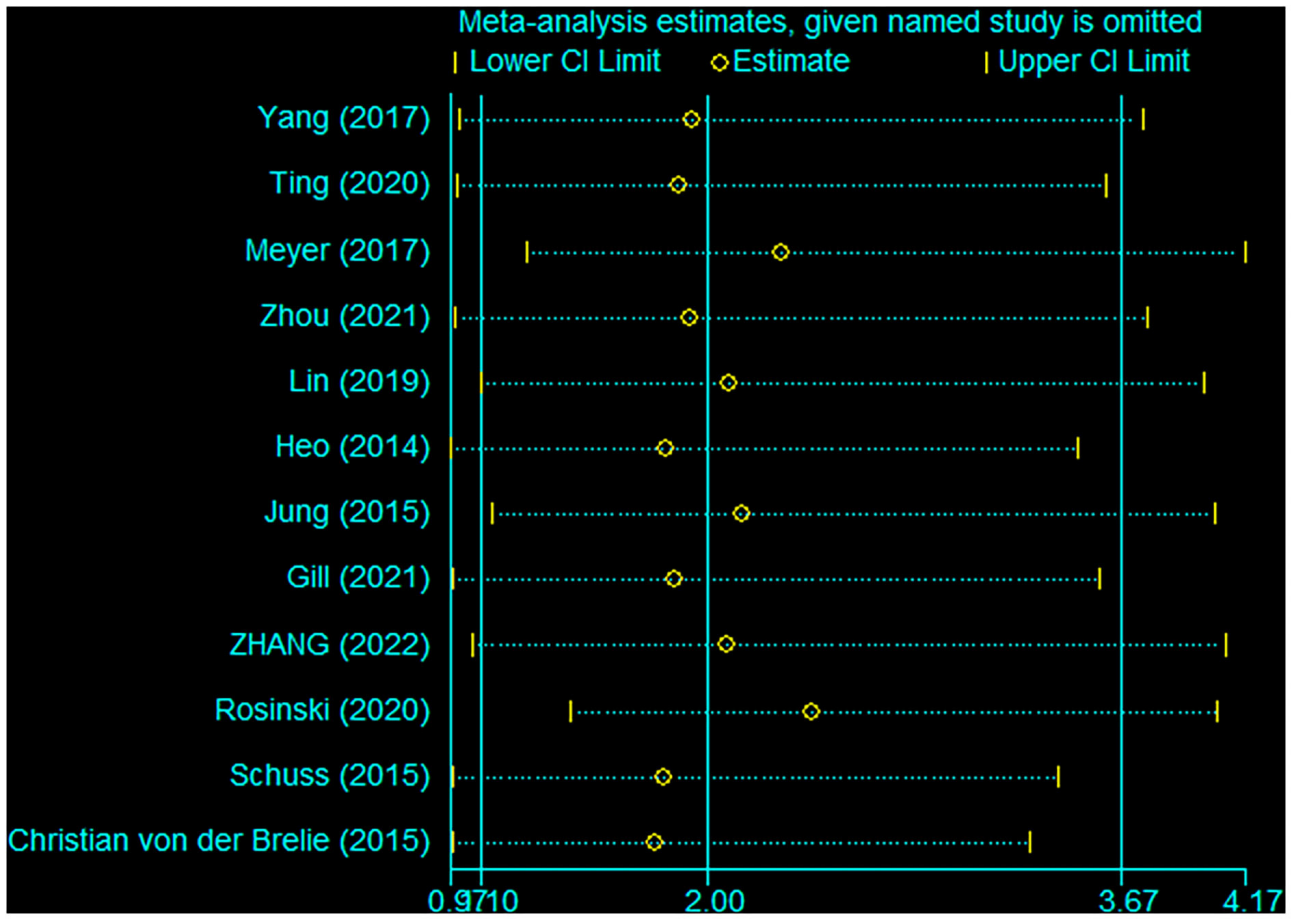
Sensitivity analysis of overall complications after staged and concurrent cranioplasty and ventriculoperitoneal shunts.

**Figure 4:**
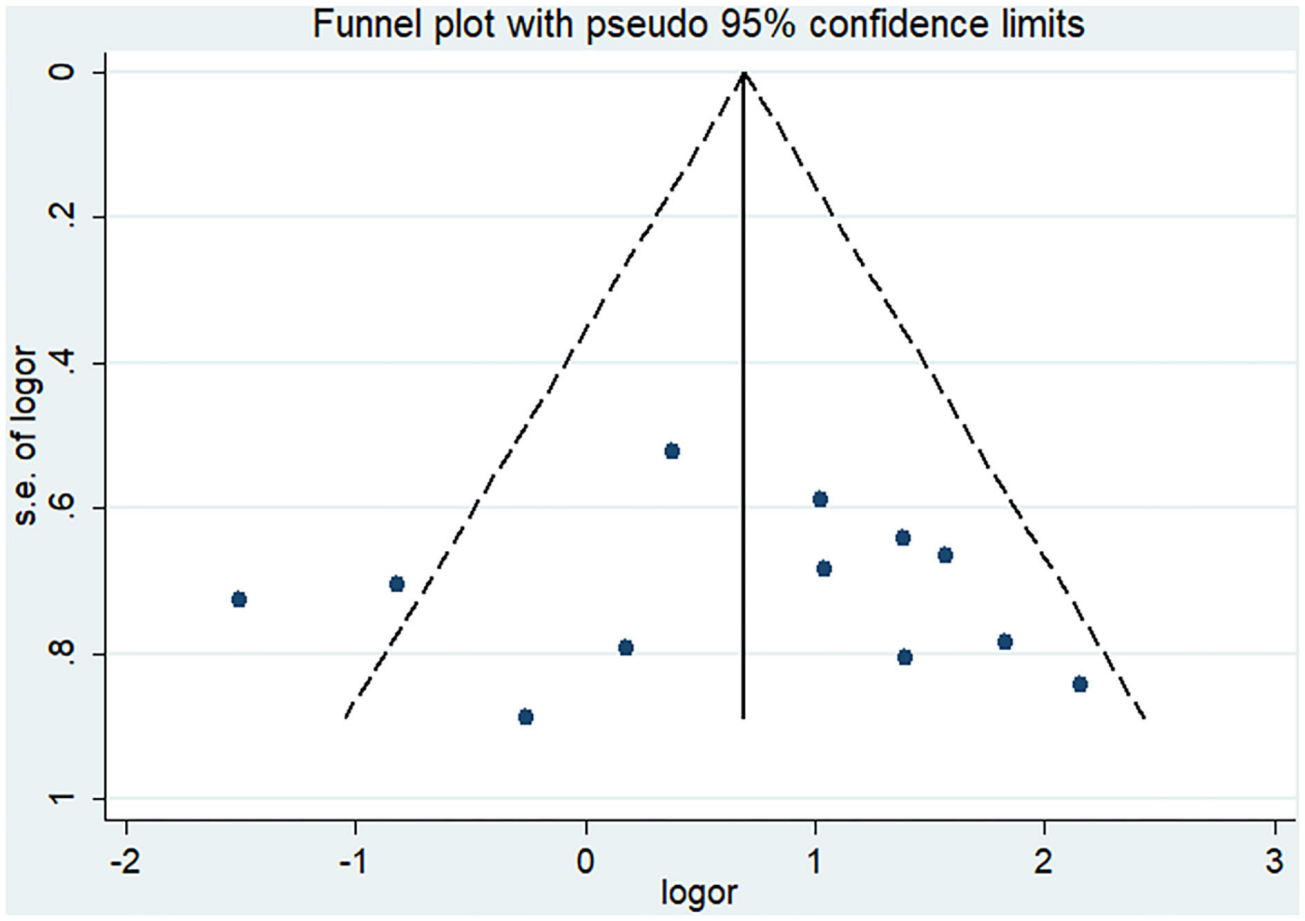
Funnel plot of overall complications after staged and concurrent cranioplasty and ventriculoperitoneal shunts.

**Figure 5:**
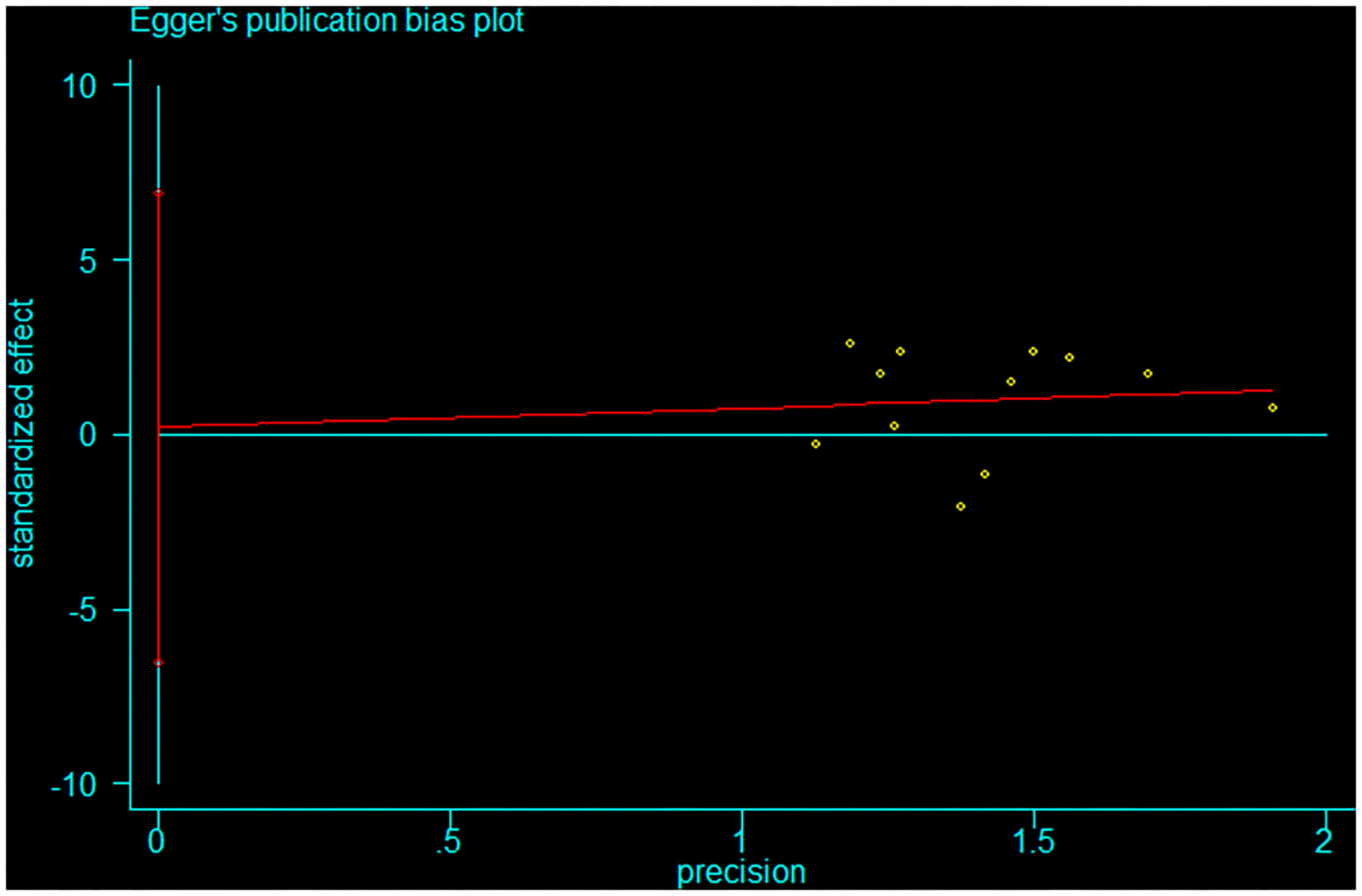
Egger trial for overall complications after staged and concurrent cranioplasty and ventriculoperitoneal shunts.

Pooled analysis of all 12 trials revealed that simultaneous cranioplasty and ventriculoperitoneal shunts resulted in greater overall complications, relative to staged surgery (pooled OR: 2.00, 95% CI: 1.10–3.67), with significant between-study heterogeneity (*I*^2^ = 56.1%; Figure 2). Ethnic subgroup analysis showed that the overall complication rate was higher in the concurrent surgical group involving the Asian ethnic group, and there was no significant heterogeneity between groups (pooled OR: 2.41, 95% CI: 1.51–3.83; *I*^2^ = 0.0%). In the Caucasian ethnic group, there were no between-group differences in overall complication rates while between-study heterogeneity was significant (pooled OR: 1.46, 95% CI: 0.24–8.77; *I*^2^ = 82.6%). After deleting any of the studies, sensitivity analysis did not reveal any significant differences, suggesting that our findings are stable (Figure 3). Funnel plot (Figure 4) and Egger trial (*p* = 0 07, Figure 5) did not show significant publication bias.

**Figure 6:**
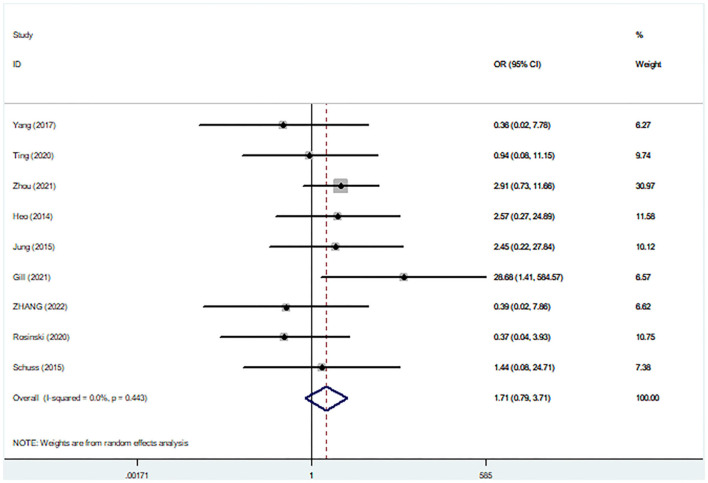
Comparisons of post-operative bleeding complications between staged and simultaneous cranioplasty and ventriculoperitoneal shunts. OR, odds ratio; CI, confidence interval.

Nine studies (*N* = 508 participants) assessed intracranial hemorrhage complication. Pooling the data of these studies did not reveal significant differences in OR of intracranial hemorrhage with concurrent surgery, compared to staged surgery (pooled OR: 1.71, 95% CI: 0.79–3.71), with low between-study heterogeneity (*I*^2^ = 0.0%; Figure 6). Ethnic subgroup analysis did not show any significant differences in OR for intracranial hemorrhage between the two surgical procedures in Asian and Caucasian groups.

**Figure 7:**
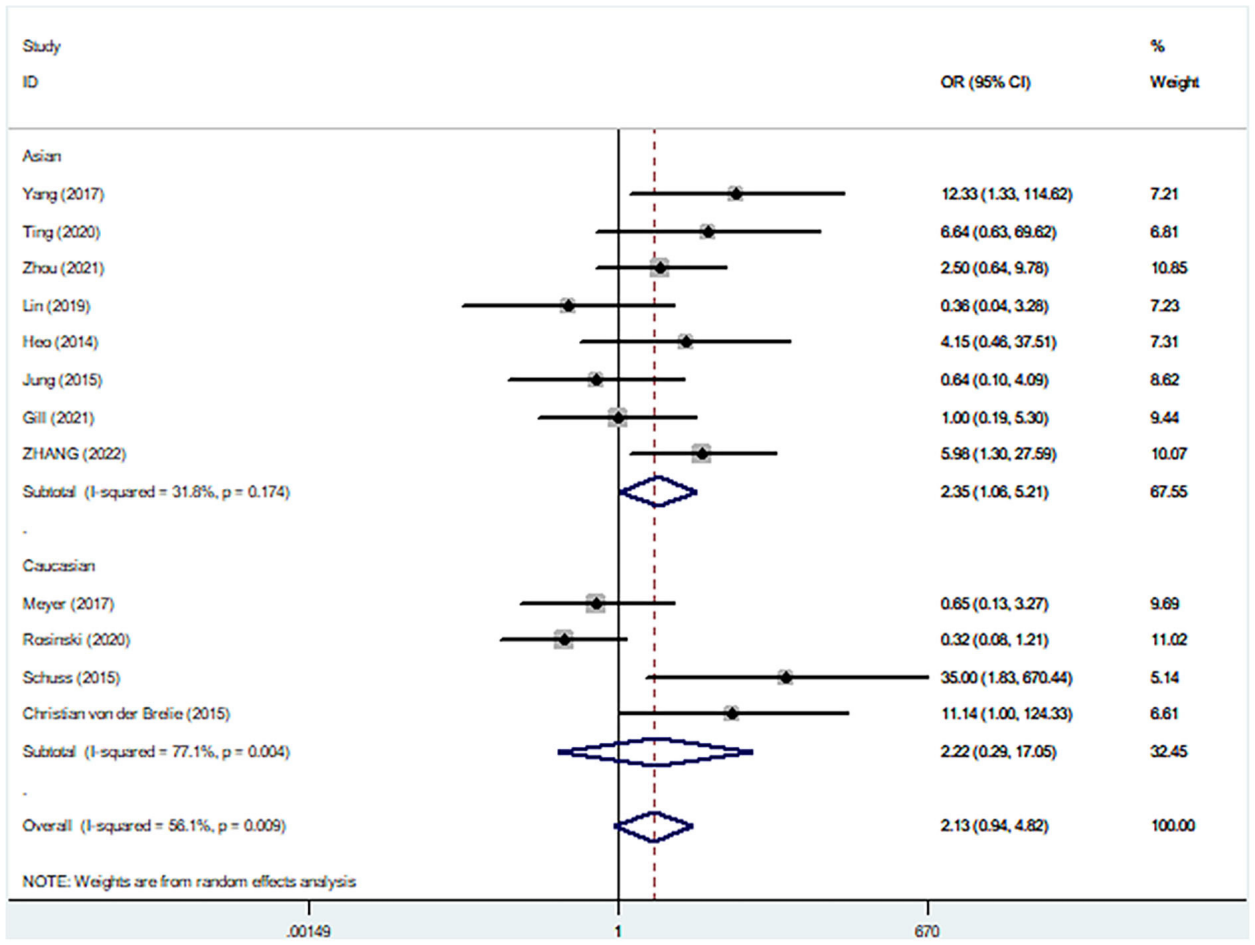
Comparisons of postoperative infection complications between staged and simultaneous cranioplasty and ventriculoperitoneal shunts.

Pooled analysis of 12 studies assessing post-operative infection complications did not reveal any significant differences in post-operative infections between the surgical approaches (pooled OR: 2.13, 95% CI: 0.94–4.82), with significant between-study heterogeneity (*I*^2^ = 56.1%; Figure 7). The funnel plot did not show any publication bias for post-surgical infections, which was supported by findings from Egger and Begg tests (all *p* = 0.064) (Supplementary Figures 1–3). Sensitivity was established to be influenced by race, and after subgroup analysis of race, the *I*^2^ value sensitivity for heterogeneity was only 31.8% for the Asian ethnicity and 77.1% for the Caucasian ethnicity. In addition, concurrent surgery of the Asian ethnicity was associated with higher incidences of postoperative infections, relative to staged surgery (pooled OR: 2.35, 95% CI: 1.06–5.21), with no significant heterogeneity between studies.

**Figure 8:**
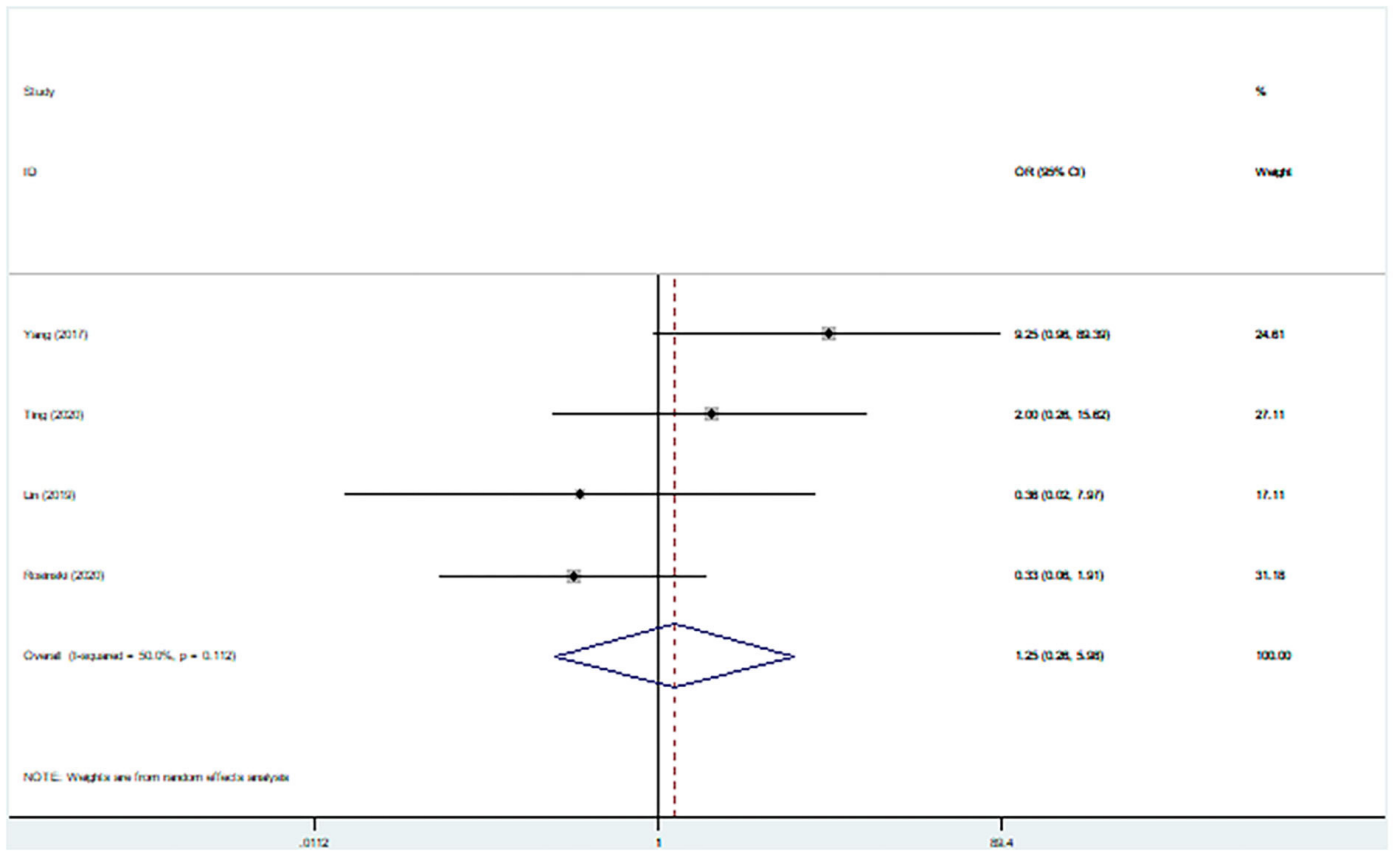
Comparisons of reoperation rates for staged and simultaneous cranioplasty and ventriculoperitoneal shunts.

Four studies (203 participants) assessed patient reoperation after treatment. Pooled data from these studies did not show any significant differences in reoperation between concurrent surgery and staged surgery groups (pooled OR: 1.25, 95% CI: 0.26–5.98), with significant between-study heterogeneity (*I*^2^ = 50.0%; Figure 8).

## Discussion

This meta-analysis of 12 articles including 651 participants showed that concurrent surgery does not increase the relative risk of post-operative bleeding, post-operative infection or re-operation compared to staged surgery; however, it increased the risk of overall complications. The only previously published meta-analysis about this topic that included seven articles showed that staged surgery significantly reduced postoperative infection, and our findings differ from theirs. In addition, we performed an innovative ethnographic subgroup analysis and concluded that concurrent surgery in the Asian population increases the relative risk of overall complications and postoperative infections, which is firstly reported.

Concurrent surgery increases the overall complication rate, compared to staged cranioplasty and ventriculoperitoneal shunts. However, differences between concurrent and staged surgery groups in terms of several specific complications, such as postoperative bleeding and infection were insignificant. Besides, reoperation rates did not differ between the groups. Ethnic subgroup analysis showed that in the Asian ethnic group, overall complication and post-operative infection rates were higher in the concurrent surgery group, compared to the staged surgery group, while differences in incidences of post-operative bleeding and re-operation complications were not marked.

Common complications when patients are subjected to cranioplasty after DC include infections, hydrocephalus, bone resorption, cerebrospinal fluid (CSF) leak, disruption of CSF flow dynamics, and non-union of the flap, with overall incidences of complications being about 40% (18). Overall complication rates for patients undergoing ventriculoperitoneal shunts after DC were reported to range from 17 to 33%, and they included intracranial hemorrhage, surgical site infection/incision healing disturbances, and shunt valve dysfunction (19, 20). Some patients with hydrocephalus after DC require both cranioplasty and ventriculoperitoneal shunt. Currently, depending on their own surgical habits and patients' requirements, clinicians choose to perform VPS placement before, during or after cranioplasty. However, there are no guidelines on staged or concurrent cranioplasty with ventriculoperitoneal shunts.

Simultaneous cranioplasty and CSF shunts have been globally performed for some years. The advantages of this approach include convenience for the patient and reduced medical costs, however, its safety has not been established. Jung et al. revealed significant correlations between higher incidences of post-operative incision site infections after concurrent surgery, relative to staged surgery. However, a ten-year retrospective analysis of concurrent vs. staged ventriculoperitoneal Shunt and cranioplasty did not reveal significant differences in terms of infection, reoperation, and resorption between the two surgical approaches, and a significantly lower incidence of hospital-acquired infections for patients in the concurrent surgery group.

Only one meta-analysis has compared the complication rates between concurrent and staged cranioplasty and ventriculoperitoneal shunts. In their meta-analysis, which involved seven articles published between 2013 and 2017, Jung et al. found that staged surgery was significantly associated with lower rates of postoperative surgical site infections, compared to concurrent surgery, but there were no differences in symptomatic intracranial hemorrhage. Their findings were comparable to ours in terms of symptomatic intracranial hemorrhage, but differed in terms of post-surgical infection rates. According to our meta-analysis, differences in intracranial hemorrhage, infection, and reoperation incidences between simultaneous and staged groups were insignificant, however, a higher overall complication rate was found in the simultaneous group. These differences may be attributed to various factors. First, we did not include the Yang et al. study because it was not a comparative study of postoperative complications after staged vs. concurrent surgery (21). Second, the Jung et al. study was published 2 years ago and several new relevant studies have been published since then, which were included in our analyses. Subsequently, our subgroup analysis revealed that higher overall complication and post-operative infection rates in the concurrent surgical group involving participants of the Asian ethnicity, compared to staged surgical group. These findings are in tandem with findings from a previous study by Jung et al.

Compared to staged surgery, simultaneous surgery is more acceptable and less costly for patients, however, safety is a major concern. Both cranioplasty and cerebrospinal fluid shunts involve the implantation of heterogeneous materials and infections may eventually lead to failure of the operation and the need for the material to be removed (22, 23). We found that simultaneous surgery increased post-operative infections in the Asian population. Given the serious consequences of surgical infections that are associated with foreign body implant surgery, concurrent surgery should be cautiously performed. Although concurrent surgery did not increase the relative risk of postoperative bleeding, postoperative infection or reoperation in the total population, the overall complication rate was increased. Considering the overall complication rate and patient safety aspects, staged surgery is more advantageous.

**Figure 9:**
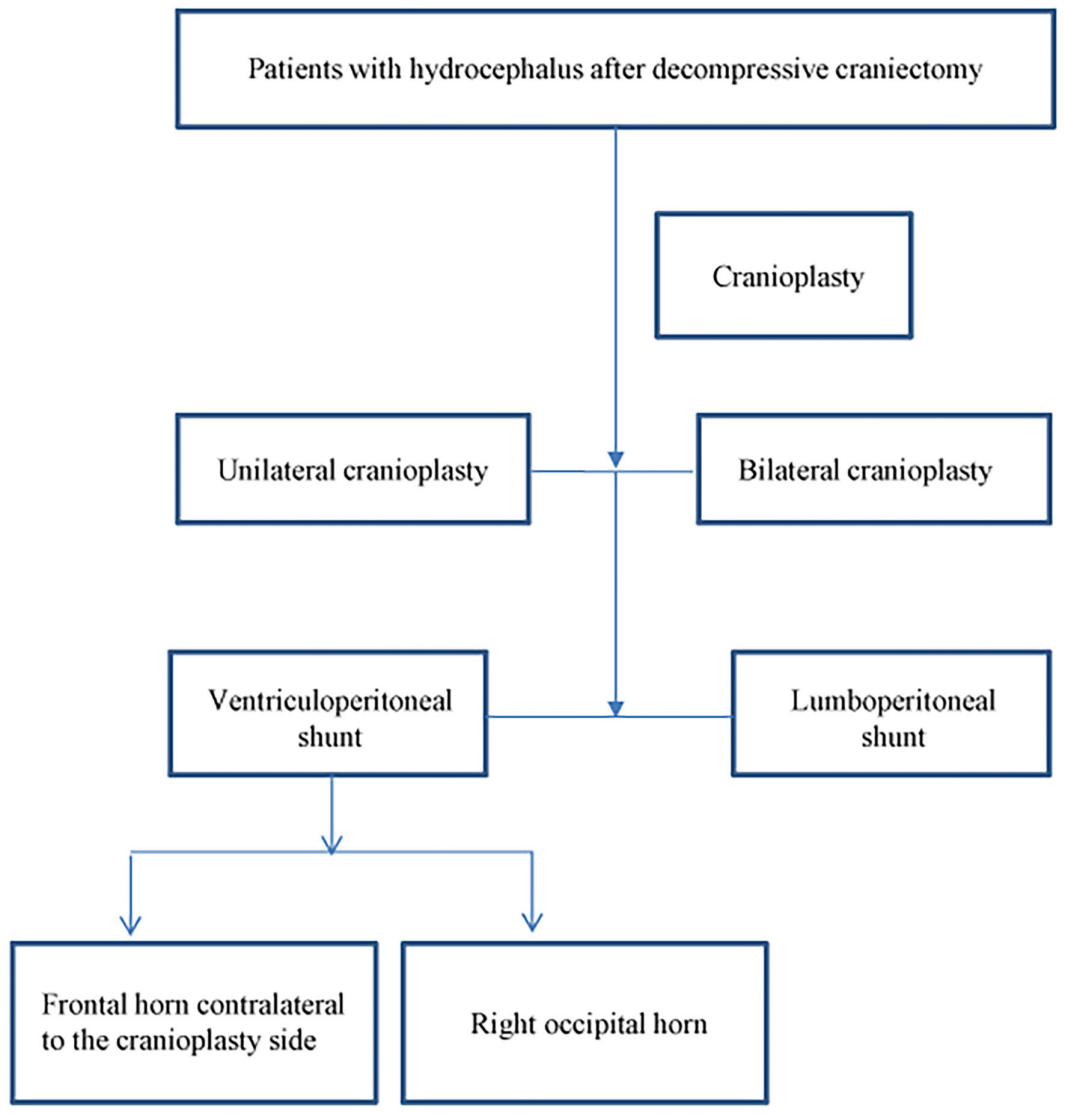
Clinical treatment flow chart for patients with hydrocephalus after decompressive craniectomy.

Besides the consideration of safety, the choice of the shunt valve is an important aspect when performing simultaneous cranioplasty and CSF shunt implantation. Changes in intracranial pressure and CSF circulation after cranioplasty have not been fully established. They have the ability to affect the choice of the shunt valve, especially whether the anti-siphon valve should be used. Currently, the programmable valve is the first choice for the CSF shunt procedure; but weather anti-siphon valve should be used is still an important factor, especially when we treat patients with normal pressure hydrocephalus. Our surgical team prefers first performing cranioplasty, and when performing consecutive shunt implantation, the shunt valve and shunt methods are chosen according to intracranial pressure and CSF circulation after cranioplasty. Commonly, we would choose to puncture frontal horn contralateral to the cranioplasty side when performing following VP shunt; and puncture right occipital horn if the patient had bilateral frontal-temporal-parietal cranioplasty. In fact, we have now performed more lumboperitoneal shunt operations. The clinical treatment flow chart is shown in Figure 9.

This meta-analysis has four limitations. First, the studies we included were retrospective observational single center studies, and their level of evidence was not as high as that of randomized controlled studies. Second, significant heterogeneity was observed in this study. However, subgroup analysis revealed that ethnicity only partially explained the source of heterogeneity. The different intervals between staged cranioplasty and ventriculoperitoneal shunts, the choice of different shunt valves in ventriculoperitoneal shunts and the different proportions of autologous bone grafts used in cranioplasty for the included studies may have contributed to the heterogeneity. Third, we excluded the pediatric population because hydrocephalus is a different disease in the adult and pediatric populations and the choice of treatment and repair material for cranioplasty is also different (24, 25). Finally, we only included studies that were relevant in English, which may have influenced our results.

## Conclusion

Overall complications were higher in the concurrent surgery group, compared to staged cranioplasty and ventriculoperitoneal shunt. However, differences between the concurrent and staged groups in terms of incidences of bleeding, infection and reoperation were insignificant, although a trend toward higher rates was observed in the concurrent group. Subgroup analysis showed that overall complications and postoperative infection rates were higher in the concurrent surgery group, relative to staged surgery group involving the Asian ethnic group. Differences in incidences of postoperative bleeding and reoperation were insignificant. More randomized, prospective trials should be performed to compare the complications associated with concurrent and staged cranioplasty and ventriculoperitoneal shunts.

## Data availability statement

The raw data supporting the conclusions of this article will be made available by the authors, without undue reservation.

## Author contributions

All authors listed have made a substantial, direct, and intellectual contribution to the work and approved it for publication.

## Funding

This research was funded by the Zhejiang Provincial Natural Science Foundation of China (No. LGF21H090010) and the National Natural Science Foundation of China (No. 81971159).

## Conflict of interest

The authors declare that the research was conducted in the absence of any commercial or financial relationships that could be construed as a potential conflict of interest.

## Publisher's note

All claims expressed in this article are solely those of the authors and do not necessarily represent those of their affiliated organizations, or those of the publisher, the editors and the reviewers. Any product that may be evaluated in this article, or claim that may be made by its manufacturer, is not guaranteed or endorsed by the publisher.
