# Supplementary material for: Is simultaneous cranioplasty with cerebrospinal fluid shunts implantation as safe as staged procedures?
Source: Front Neurol. 2022 Oct 10;13:995897. doi: 10.3389/fneur.2022.995897 (PMC9588942; doi:10.3389/fneur.2022.995897)
Supplement: Supplementary file 2 [file Presentation_1.zip › Supplementary material/Supplemental figure legends.docx]

Supplementary Fig. 1 Funnel plot of post-operative infection complications after staged and concurrent cranioplasty and ventriculoperitoneal shunts.

Supplementary Fig. 2 Egger trial for post-operative infection complications after staged and concurrent cranioplasty and ventriculoperitoneal shunts.

Supplementary Fig. 3 Begg’s funnel plot of post-operative infection complications after staged and concurrent cranioplasty and ventriculoperitoneal shunts.
