# Supplementary material for: Is simultaneous cranioplasty with cerebrospinal fluid shunts implantation as safe as staged procedures?
Source: Front Neurol. 2022 Oct 10;13:995897. doi: 10.3389/fneur.2022.995897 (PMC9588942; doi:10.3389/fneur.2022.995897)
Supplement: Supplementary file 2 [file Presentation_1.zip › Supplementary material/supplemental search strategy.docx]

Search strategy for each database.

Search date 2022-5-11

**Pubmed:**

Total number: 122

Search: ((("Ventriculoperitoneal Shunt"[Mesh]) OR ((((((((Shunt, Ventriculoperitoneal) OR (Shunts, Ventriculoperitoneal)) OR (Ventriculoperitoneal Shunts)) OR (Ventriculo-peritoneal Shunt)) OR (Shunt, Ventriculo-peritoneal)) OR (Shunts, Ventriculo-peritoneal)) OR (Ventriculo peritoneal Shunt)) OR (Ventriculo-peritoneal Shunts))) AND ((Cranial vault reconstruction) OR (Cranioplasty))) AND (((((((Craniectomy, Decompressive) OR (Decompressive Craniectomies)) OR (Decompressive Craniotomy)) OR (Craniotomy, Decompressive)) OR (Decompressive Craniotomies)) OR ("Decompressive Craniectomy"[Mesh])) OR (("Hydrocephalus"[Mesh]) OR (((((((((((((((((((((((((((Hydrocephaly) OR (Cerebral Ventriculomegaly)) OR (Cerebral Ventriculomegalies)) OR (Ventriculomegalies, Cerebral)) OR (Ventriculomegaly, Cerebral)) OR (Communicating Hydrocephalus)) OR (Hydrocephalus, Communicating)) OR (Congenital Hydrocephalus)) OR (Hydrocephalus, Congenital)) OR (Hydrocephalus Ex-Vacuo)) OR (Hydrocephalus Ex Vacuo)) OR (Hydrocephalus Ex-Vacuos)) OR (Obstructive Hydrocephalus)) OR (Hydrocephalus, Obstructive)) OR (Post-Traumatic Hydrocephalus)) OR (Hydrocephalus, Post-Traumatic)) OR (Post Traumatic Hydrocephalus)) OR (Aqueductal Stenosis)) OR (Aqueductal Stenoses)) OR (Stenoses, Aqueductal)) OR (Stenosis, Aqueductal)) OR (Fetal Cerebral Ventriculomegaly)) OR (Cerebral Ventriculomegalies, Fetal)) OR (Cerebral Ventriculomegaly, Fetal)) OR (Fetal Cerebral Ventriculomegalies)) OR (Ventriculomegalies, Fetal Cerebral)) OR (Ventriculomegaly, Fetal Cerebral))))

**Embase:**

#1 'decompressive craniectomy'/exp

#2 'craniectomy, decompressive' OR 'decompressive craniectomies' OR 'decompressive craniotomy'/exp OR 'decompressive craniotomy' OR 'craniotomy, decompressive' OR 'decompressive craniotomies'

#3 #1 OR #2

#4 'hydrocephalus'/exp

#5 hydrocephaly OR 'cerebral ventriculomegaly' OR 'cerebral ventriculomegalies' OR 'ventriculomegalies, cerebral' OR 'ventriculomegaly, cerebral' OR 'communicating hydrocephalus' OR 'hydrocephalus, communicating' OR 'congenital hydrocephalus' OR 'hydrocephalus, congenital' OR 'hydrocephalus ex-vacuo' OR 'hydrocephalus ex vacuo' OR 'hydrocephalus ex-vacuos' OR 'obstructive hydrocephalus' OR 'hydrocephalus, obstructive' OR 'post-traumatic hydrocephalus' OR 'hydrocephalus, post-traumatic' OR 'post traumatic hydrocephalus' OR 'aqueductal stenosis' OR 'aqueductal stenoses' OR 'stenoses, aqueductal' OR 'stenosis, aqueductal' OR 'fetal cerebral ventriculomegaly' OR 'cerebral ventriculomegalies, fetal' OR 'cerebral ventriculomegaly, fetal' OR 'fetal cerebral ventriculomegalies' OR 'ventriculomegalies, fetal cerebral' OR 'ventriculomegaly, fetal cerebral'

#6 #4 OR #5

#7 #3 OR #6

#8 'brain ventricle peritoneum shunt'/exp

#9 'cerebrospinal fluid shunting'/exp

#10 'shunt, ventriculoperitoneal' OR 'shunts, ventriculoperitoneal' OR 'ventriculoperitoneal shunts' OR 'ventriculo-peritoneal shunt' OR 'shunt, ventriculo-peritoneal' OR 'shunts, ventriculo-peritoneal' OR 'ventriculo peritoneal shunt' OR 'ventriculo-peritoneal shunts'

#11 #8 OR #9 OR #10

#12 'cranioplasty'/exp

#13 'cranial vault reconstruction'

#14 #12 OR #13

#15 #11 AND #14

#16 #7 AND #15

**Cochrane Library**

Total number:1

#1 MeSH descriptor: [Decompressive Craniectomy] explode all treesMeSH

#2 MeSH descriptor: [Hydrocephalus] explode all treesMeSH

#3((Craniectomy, Decompressive)) OR ((Decompressive Craniectomies)) OR ((Decompressive Craniotomy)) OR ((Craniotomy, Decompressive)) OR ((Decompressive Craniotomies))

#4 #1 OR #3

#5((Hydrocephaly)) OR ((Cerebral Ventriculomegaly)) OR ((Cerebral Ventriculomegalies)) OR ((Ventriculomegalies, Cerebral)) OR ((Ventriculomegaly, Cerebral))

#6((Communicating Hydrocephalus)) OR ((Hydrocephalus, Communicating)) OR ((Congenital Hydrocephalus)) OR ((Hydrocephalus, Congenital)) OR ((Hydrocephalus Ex-Vacuo))

#7((Hydrocephalus Ex Vacuo)) OR ((Hydrocephalus Ex-Vacuos)) OR ((Obstructive Hydrocephalus)) OR ((Hydrocephalus, Obstructive)) OR ((Post-Traumatic Hydrocephalus))

#8((Hydrocephalus, Post-Traumatic)) OR ((Post Traumatic Hydrocephalus)) OR ((Aqueductal Stenosis)) OR ((Aqueductal Stenoses)) OR ((Stenoses, Aqueductal))

#9((Stenosis, Aqueductal)) OR ((Fetal Cerebral Ventriculomegaly)) OR ((Cerebral Ventriculomegalies, Fetal)) OR ((Cerebral Ventriculomegaly, Fetal)) OR ((Fetal Cerebral Ventriculomegalies))

#10((Ventriculomegalies, Fetal Cerebral)) OR ((Ventriculomegaly, Fetal Cerebral))

#11#2 OR #5 OR #6 OR #7 OR #8 OR #9 OR #10

#12 MeSH descriptor: [Ventriculoperitoneal Shunt] explode all trees MeSH

#13 ((Shunt, Ventriculoperitoneal)) OR ((Shunts, Ventriculoperitoneal)) OR ((Ventriculoperitoneal Shunts)) OR ((Ventriculo-peritoneal Shunt)) OR ((Shunt, Ventriculo-peritoneal))

#14 ((Shunts, Ventriculo-peritoneal)) OR ((Ventriculo peritoneal Shunt)) OR ((Ventriculo-peritoneal Shunts))

#15 #12 OR #13 OR #14

#16((Cranioplasty)) OR ((Cranial vault reconstruction))

#17 #4 OR #11

#18 #17 AND #15 AND #16

**Web of Science**

total number:145

#1 Decompressive Craniectomy or （Craniectomy, Decompressive）or （Decompressive Craniectomies） or （Decompressive Craniotomy） or （Craniotomy, Decompressive）or （Decompressive Craniotomies）

#2 Hydrocephalus or Hydrocephaly or （Cerebral Ventriculomegaly）or （Cerebral Ventriculomegalies） or （Craniotomy, Decompressive） or （Ventriculomegalies, Cerebral） or （Ventriculomegaly, Cerebral） or （Communicating Hydrocephalus） or （Hydrocephalus, Communicating） or （Congenital Hydrocephalus） or （Hydrocephalus, Congenital） or （Hydrocephalus Ex-Vacuo） or （Hydrocephalus Ex Vacuo） or （Hydrocephalus Ex-Vacuos） or （Obstructive Hydrocephalus） or （Hydrocephalus, Obstructive） or （Post-Traumatic Hydrocephalus） or （Hydrocephalus, Post-Traumatic）or （Post Traumatic Hydrocephalus） or （Aqueductal Stenosis） or （Aqueductal Stenoses） or （Stenoses, Aqueductal） or （Stenosis, Aqueductal） or （Fetal Cerebral Ventriculomegaly） or （Cerebral Ventriculomegalies, Fetal） or （Cerebral Ventriculomegaly, Fetal） or （Fetal Cerebral Ventriculomegalies） or （Ventriculomegalies, Fetal Cerebral） or （Ventriculomegaly, Fetal Cerebral）

#3（Ventriculoperitoneal Shunt） or （Shunt, Ventriculoperitoneal）or （Shunts, Ventriculoperitoneal） or （Ventriculoperitoneal Shunts）or （Ventriculo-peritoneal Shunt） or （Shunt, Ventriculo-peritoneal）or （Shunts, Ventriculo-peritoneal） or （Ventriculo peritoneal Shunt） or （Ventriculo-peritoneal Shunts）

#4（Cranioplasty） or （Cranial vault reconstruction）

#5 #4 AND #3

#6 #1 OR #2

#7 #5 AND #6
