# Supplementary figures and images for: Is simultaneous cranioplasty with cerebrospinal fluid shunts implantation as safe as staged procedures?
Source: Front Neurol. 2022 Oct 10;13:995897. doi: 10.3389/fneur.2022.995897 (PMC9588942; doi:10.3389/fneur.2022.995897)

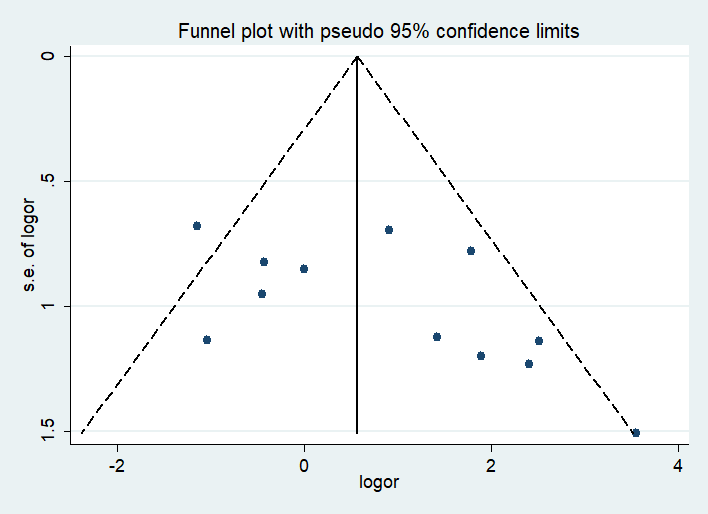

Supplement: Supplementary file 2 [file Presentation_1.zip › Supplementary material/supplementary Fig.1.tif]

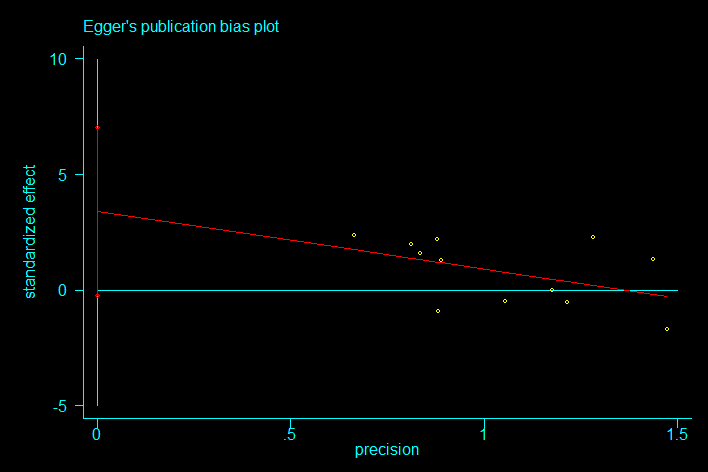

Supplement: Supplementary file 2 [file Presentation_1.zip › Supplementary material/supplementary Fig.2.tif]

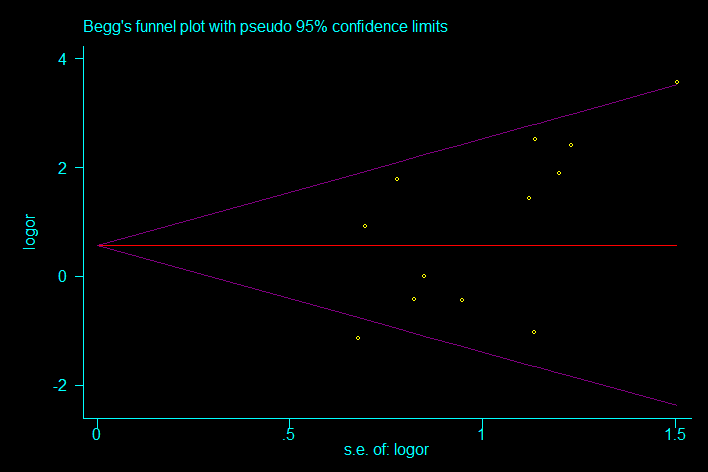

Supplement: Supplementary file 2 [file Presentation_1.zip › Supplementary material/supplementary Fig.3.tif]
